# Publisher’s Note: Phage treatment of an aortic graft infected with *Pseudomonas aeruginosa*

**DOI:** 10.1093/emph/eoz006

**Published:** 2019-03-04

**Authors:** Benjamin K Chan, Paul E Turner, Samuel Kim, Hamid R Mojibian, John A Elefteriades, Deepak Narayan

**Affiliations:** 1 Department of Ecology and Evolutionary Biology, Yale University, New Haven, CT, USA; 2 Program in Microbiology, Yale School of Medicine, New Haven, CT, USA; 3 Section of Plastic and Reconstructive Surgery, Department of Surgery, Yale School of Medicine, New Haven, CT, USA; 4 Department of Radiology and Biomedical Imaging, Yale School of Medicine, New Haven, CT, USA; 5 Section of Cardiac Surgery, Department of Surgery, Yale School of Medicine, New Haven, CT, USA

The corresponding author for this article is now deceased, and the corresponding author information has since been changed from Deepak Narayan to Paul E. Turner.

